# NMR metabolomic profiling of cerebrospinal fluid from dogs with meningoencephalitis of unknown origin demonstrates metabolic similarities to multiple sclerosis

**DOI:** 10.1007/s11306-026-02403-x

**Published:** 2026-02-09

**Authors:** Rita Gonçalves, Gemma Walmsley, Thomas W. Maddox, Emily J. Clarke, Marie M. Phelan

**Affiliations:** 1 Department of Veterinary Science, Small Animal Teaching Hospital, University of Liverpool, Leahurst, Neston, Cheshire CH64 7TE UK; 2 Department of Musculoskeletal and Ageing Science, Institute of Lifecourse and Medical Sciences, University of Liverpool, Liverpool, UK; 3 Highfield NMR Facility, Liverpool Shared Research Facilities, Faculty of Health and Lifesciences, University of Liverpool, Liverpool, UK

**Keywords:** Canine, MUO, CSF, Metabolomics, 1H NMR

## Abstract

**Introduction:**

Meningoencephalitis of unknown origin (MUO) in dogs is a debilitating and often fatal disease that shows similarities to multiple sclerosis (MS) in humans. The metabolomic profile of MUO has not been previously reported.

**Objectives:**

To compare the metabolomic profile of cerebrospinal fluid (CSF) of dogs with MUO with two other diseases affecting the central nervous system in dogs, steroid responsive meningitis-arteritis (SRMA) and idiopathic epilepsy (IE), and to determine if the metabolic profile of MUO shows similarities with that of MS.

**Methods:**

Untargeted and semi-targeted metabolomics using ^1^H nuclear magnetic resonance (NMR) was performed on surplus CSF of dogs diagnosed with MUO, SRMA and IE. Data were examined by multivariate and univariate statistical analysis and pathway analysis.

**Results:**

Fifty-six metabolites were identified in 56 dogs. The multivariate analysis of the canine data highlighted significant differences between the different disease groups. Most metabolites were increased in SRMA and decreased in IE when compared to MUO. Most affected metabolites included those involved in energy metabolism. Pathway analysis revealed that these metabolites were mainly involved in pyruvate metabolism, glycolysis or gluconeogenesis, glycine, serine and threonine metabolism and alanine, aspartate and glutamate metabolism.

**Conclusion:**

These results suggest that there is an increased energy demand in MUO. Our findings provide a first-time overview of CSF metabolic changes in MUO and offer potential insights for possible underlying pathogenesis and treatment strategies. Altered energy metabolism pathways are also reported in MS, further supporting the use of MUO as a spontaneous animal model for this disease.

**Supplementary Information:**

The online version contains supplementary material available at 10.1007/s11306-026-02403-x.

## Introduction

Canine meningoencephalitis of unknown origin (MUO) is a heterogeneous disorder that encompasses several idiopathic inflammatory diseases, including granulomatous meningoencephalomyelitis (GME), necrotizing meningoencephalomyelitis (NME) and necrotizing leucoencephalitis (NLE) (Cornelis et al., 2019; Granger et al., 2010; Nessler & Tipold, 2024; Talarico & Schatzberg, 2010). These conditions can only be conclusively diagnosed by histopathological evaluation of the brain based on their unique characteristics, although significant overlap can be recognized in some cases (Nessler et al., 2022). The clinical diagnosis of MUO typically relies on identification of suggestive imaging and clinicopathological findings but, to date, there are no universally established diagnostic criteria due to the heterogeneity of the disease. The incidence of MUO in the canine population is unknown but, in UK referral populations, MUO is thought be the most common inflammatory condition affecting the central nervous system (CNS) in dogs (Gonçalves et al., 2021).

Despite initiation of appropriate treatment, 25–33% of dogs with MUO are reported to die within one week of diagnosis (Cornelis et al., 2019) and 36.5% within 6 months of diagnosis (Gonçalves et al., 2024b). Unfortunately, even for those patients that survive, residual neurological deficits are common and likely affect the dog’s quality of life (Cornelis et al., 2019; Gonçalves et al., 2024b; Granger et al., 2010). Clinical and imaging findings are variable making the study and treatment of MUO challenging (Cornelis et al., 2019; Gonçalves et al., 2024a; Talarico & Schatzberg, 2010). Although the pathogenesis is poorly understood, current evidence suggests that MUO arises in genetically susceptible dogs after exposure to as yet undetermined environmental triggers (Nessler & Tipold, 2024). A better understanding of the biological characteristics of MUO would therefore be very useful to provide insights into the pathogenesis and mechanisms of this debilitating disease. Several studies have noted similarities between MUO (particularly the NME form) and multiple sclerosis (MS) in humans (Greer et al., 2010; Jeffery & Granger, 2023; Prümmer et al., 2023) and proposed MUO as a model this condition.

Suitable biofluids for investigation of potential biomarkers in diseases of the CNS include the cerebrospinal fluid (CSF), plasma and urine. CSF has the benefit of greater proximity to the site of neuropathology in the brain and reflecting the biochemical environment of the CNS (Zhang et al., 2013). Metabolic profiling of CSF reveals a large number of detectable and quantifiable metabolites and is therefore potentially useful in the study of systems biology and discovery of biomarkers with clinical applications (Rosenling et al., 2011). One of its main advantages is that it reflects the actual state of the CNS during inflammation and, due to the small concentrations of proteins within it, does not need significant preparation before laboratory analyses. Identifying biomarkers for neurological disease such as MUO is particularly important given the limitations in accessing the CNS in vivo and could result in more efficient treatment and lower mortality in this condition. Metabolomics analysis of CSF in several CNS diseases in humans has identified possible biomarkers and shown usefulness in increasing diagnostic accuracy and improving patient outcomes (Zhang et al., 2013). Using a semi-targeted metabolomics approach, the identification and quantification of metabolites could help generate a disease profile for MUO and discriminate patients with MUO from other diseases. Determination of the differential metabolites might also facilitate the understanding of the pathophysiology of MUO, currently still poorly understood.

The main aim of our study was to analyze CSF samples from dogs with MUO and compare those with other common CNS conditions, i.e. idiopathic epilepsy (IE) and steroid responsive meningitis-arteritis (SRMA), using ^1^H-NMR and determine if metabolomics profiling could distinguish between these diseases. We also aimed to determine if the metabolic profile of MUO in dogs shows similarities with that of MS in humans.

## Methods

### Study design and patient population

This study was a single center prospective case-control study. Surplus CSF samples from dogs diagnosed with meningoencephalitis of unknown origin (MUO), idiopathic epilepsy (IE) and steroid responsive meningitis-arteritis (SRMA) were collected at the Small Animal Teaching Hospital of the University of Liverpool between June 2020 and December 2023. The demographic and clinical data recorded for all dogs included sex, age, date and site of CSF collection (cervical or lumbar).

Dogs meeting the following criteria were diagnosed with MUO: 1) clinical examination consistent with focal or multifocal intracranial neuroanatomical localization; 2) older than 6 months of age; 3) multiple, single or diffuse intra-axial hyperintensities on T2-weighted magnetic resonance imaging (MRI) (Nessler & Tipold, 2024). Cerebrospinal fluid pleocytosis was not required for inclusion in this study due to previous studies showing that it can be normal in a significant proportion of dogs with MUO (Flegel, 2017; Granger et al., 2010; Talarico & Schatzberg, 2010) but the results were recorded.

Dogs were diagnosed with IE if they had suffered at least two seizures (more than 24 h apart), had a normal neurological examination, normal MRI of the brain and normal CSF analysis, *corresponding to a Tier II confidence level for the diagnosis of IE according to the International Veterinary Epilepsy Task Force *(De Risio, et al., 2015). Dogs were diagnosed with SRMA if they presented with typical clinical signs (pyrexia and spinal pain), had normal imaging of the cervical spine (radiography, computed tomography and/or MRI) and mixed or neutrophilic pleocytosis on CSF analysis (Wohlsein & Tipold, 2023).

### Sample preparation and metabolite identification

CSF was collected by either cervical or lumbar collection, based on clinician preference or clinical indications for diagnostic purposes. Surplus CSF was then immediately centrifuged at *8000 x* g for 3 min (to remove red and white blood cells) and then stored at − 80 °C until the NMR experiment. Samples were then thawed and 180 µl of CSF sample mixed with 20 µl stock solution of NMR buffer containing 100 mM sodium phosphate buffer and 0.1 mM of trimethylsilyl-propanoic acid (TSP). Samples were vortexed for 1 min and centrifuged at 4 °C for 5 min at 21,500 x g and 200 µl transferred into a 3 mm outer diameter NMR tube using a glass pipette.

To identify metabolites, ^1^H NMR spectra were acquired on a 700 MHz NMR Bruker Avance IIIHD spectrometer equipped with a TCI cryoprobe and chilled SampleJet™ Autosampler. Spectrometer set-up prior to acquisition of each batch followed the quality assurance criteria set out by the Metabolomics Standards Initiative (MSI) (Salek et al.,^2013^; Sumner et al., 2007). Briefly, spectrometer probe temperature calibrated to 25 °C +/ − 0.1 °C and three-dimensional shimming meeting line width quality established at probe acceptance. Temperature was calibrated via comparison to Bruker standard 99.8 deuterated methanol thermometer (catalogue number Z10627, Bruker UK) (Findeisen et al., 2007). Three-dimensional shimming calibrated via 3D shim routine using vendor supplied ‘topshim’ routine on Bruker aqueous Sucrose and DSS standard in 10% ^2^H_2_O 90% ^1^H_2_O (catalogue Z10902 number, Bruker UK) with acceptance criteria of *line width* half height for DSS < 1Hz.

CSF ^1^H spectra were acquired with vendor supplied pulse sequence 1D Carr Purcell Meiboom Gill (CPMG) pulse sequence for attenuation of any large molecules (key parameters described in Table S1). Acquired data were automatically processed (Fourier transformation, 0.3 Hz line broadening, phasing, alignment and baseline correction) using a vendor supplied processing routine (apk0.noe). Resultant spectra were manually inspected to establish they meet recommended quality control criteria set out by the MSI (Salek et al., 2013; Sumner et al., 2007). Briefly, line width half height for glucose anomeric at 5.24ppm reference peak (TSP varying with protein was added but not consistent) within 1 standard deviation, flat baseline, residual water peak less than 0.4 ppm wide and comparable signal to noise across all spectra. All spectra was appraised for line width at half height from initial acquisition of CPMG 1Ds. The mean and standard deviation of line width half height was calculated and any spectra outside this threshold (1.43–1.97 Hz) was resubmitted for acquisition within 48 h of the original acquisition. Randomly selected samples meeting line width threshold were also resubmitted to determine whether any metabolite drift was observed after 3 repeats (within 1 week of sample preparation for NMR) all samples met inclusion threshold.

For quantitative metabolomics profiling, spectra were then annotated using Bruker TopSpin 3.5 (Bruker, UK), Chenomx NMR suite (v8,2) and in house Galaxy toolkit tameNMR (Github – https://github.com/PGB-LIV/tameNMR: Tools for Analysis of Metabolomic NMR [tameNMR]). *The spectra were matched either to the chenomx external 1D reference library (MSI level 2) or where indicated in Table S2*,* matched to the in house 2D (J-resolved*,* 1 H 1 H TOCSY and 1 H 13 C HSQC) reference standard library (MSI level 1 annotation) using* www.ccpn.ac.uk (Table S2 and Figure S1) (Skinner et al., 2016; Worley & Powers, 2016). The spectra were then divided into variable width peak “bins” *by adjusting the individual ppm boundaries for each annotation* except for the regions between 4.52 and 5.18 to remove the variations in the presaturation of the residual water ( https://github.com/PGB-LIV/tameNMR/blob/master/tameNMR/ProcessSpectra/BinSpectra.R)*.* Where multiple bins were annotated with a single metabolite, an in-house correlation reliability scoremetric (CRS) was applied to select the most representative bin for each metabolite for use in further analyses (Coope et al., 2022; Grosman, 2019). *This score was determined by calculating the Pearson correlation matrix for all bins identified per metabolite*,* extracting the individual bin correlation values and then calculating the mean correlation for each individual bin of the metabolite.* Where correlation scores were low between bins or scores were tied, bins were manually examined to determine the most representative bin. Factors in the selection process included signal-to noise ratio, presence of overlapping spectra and the Chenomx and tameNMR visualization of relevant bins.

### Data processing and statistical analysis

The spectra were classified into the three diagnostic groups (MUO, IE and SRMA). After normalisation to total area under the curve, the quantified metabolites were pareto-scaled to reduce the influence of quantity variability among the samples. The metabolites were assessed for normality using the Shapiro-Wilk test and as not all were normally distributed, non-parametric tests were chosen to evaluate the data. The Kruskal Wallis test, Wilcoxon rank-sum test, principal component analysis (PCA) and partial-least-square discriminant analysis (PLS-DA) were carried out using MetaboAnalyst (version 6.0, https://www.metaboanalyst.ca/). For the *Wilcoxon rank-sum and Kruskal Wallis tests*, correction for multiple analysis was performed using the Benjamini-Hochberg False-Discovery Rate (FDR) method. The data were first analysed by PCA to explore the sample distributions and identify potential outliers within the datasets. In addition to the Kruskal Wallis test, parametric one-way analysis of variance (ANOVA) with Tukey post hoc analysis was used determined means differences in individual metabolites between all three disease groups and pairwise univariate analysis using Wilcoxon rank-sum tests was used to identify metabolites contributing to the discrimination among groups. Supervised PLS-DA was used to build predictive models between MUO and the other disease groups. As PLS-DA are supervised discriminatory models, performance metrics were calculated to determine model robustness in accordance with best practice (Szymańska, et al., 2012). The model performance was assessed by performing multiple PLS-DA models to assess five-fold cross validation per PLS-DA, evaluating the predicted variation (Q^2^) and the explained variation (R^2^). The performance of the PLS-DA model was also conducted by confusion matrix (Dankers et al., 2019) assessing specificity, sensitivity and balanced accuracy, whereby each model built was appraised for number of true positives (correct prediction or classification) and false positives (incorrect prediction or classification) using R 4.3.2 and mix0mics package as previously described (Rohart et al., 2017).

The PLS-DA model was then further used to identify the metabolites of discriminatory potential based on their higher ranking in the VIP (variable importance on projection) score plot. he appropriate number of components used in the VIP model was determined based on the cross-validation results in MetaboAnalyst (highest predictive sum of squares Q*T*^*2*^ metric).

### Pathway analysis

Metabolite pathway analysis was performed by MetaboAnalyst 6.0, employing over representation analysis (ORA) algorithms using metabolites with a VIP value > 1.0. Based on the FDR (< 0.05) and impact value score (> 0.1), the possible target metabolic pathways were identified. The Canis lupus familiaris Kyoto Encyclopedia of Genes and Genomes (http://www.kegg.jp/) was the selected pathway library.

## Results

### Clinical characteristics of dogs and CSF metabolomic analysis

Sixty-one dogs were initially included in the study: 23 with MUO, 16 with SRMA and 22 with IE. PCA was first applied to each group of samples to explore the group differentiation. PCA analysis identified five samples (3 from the MUO group and 2 from the SRMA group) lay outside the borderline of the 95% confidence ellipse, thus these samples were excluded from further analysis. In two of those dogs (one with MUO and one with SRMA), the CSF protein concentration was above 6 g/l (normal less than 0.25 g/l or 0.4 g/l in cervical and lumbar samples respectively). Two dogs with MUO were presented in status epilepticus (with the CSF samples collected at this time) and it has been shown that status epilepticus can result in significant changes to the CSF metabolome (Hanin et al., 2024). One dog with SRMA had a chronic history of relapsing remitting clinical signs and had been administered corticosteroids prior to presentation. Clinical and demographic characteristics of the final study population (20 dogs with MUO, 14 dogs with SRMA and 22 dogs with IE) are summarized in Table S3. Median duration of clinical signs prior to CSF collection in dogs with MUO was 7 days (interquartile range 5–10).

Fifty-six metabolites were annotated to 383 peak bins – for downstream analysis a single bin per metabolite was selected (Figures S2 and S3 and Table S2). Three components (ethanol, isopropyl alcohol and mannitol) of likely exogenous origin (the first two are commonly used for skin antiseptic preparation for CSF collection and the latter was administered to the dogs with signs of raised intracranial pressure at time of CSF collection) were excluded from the statistical analysis.

### Metabolite profiles by disease groups

**Fig. 1:**
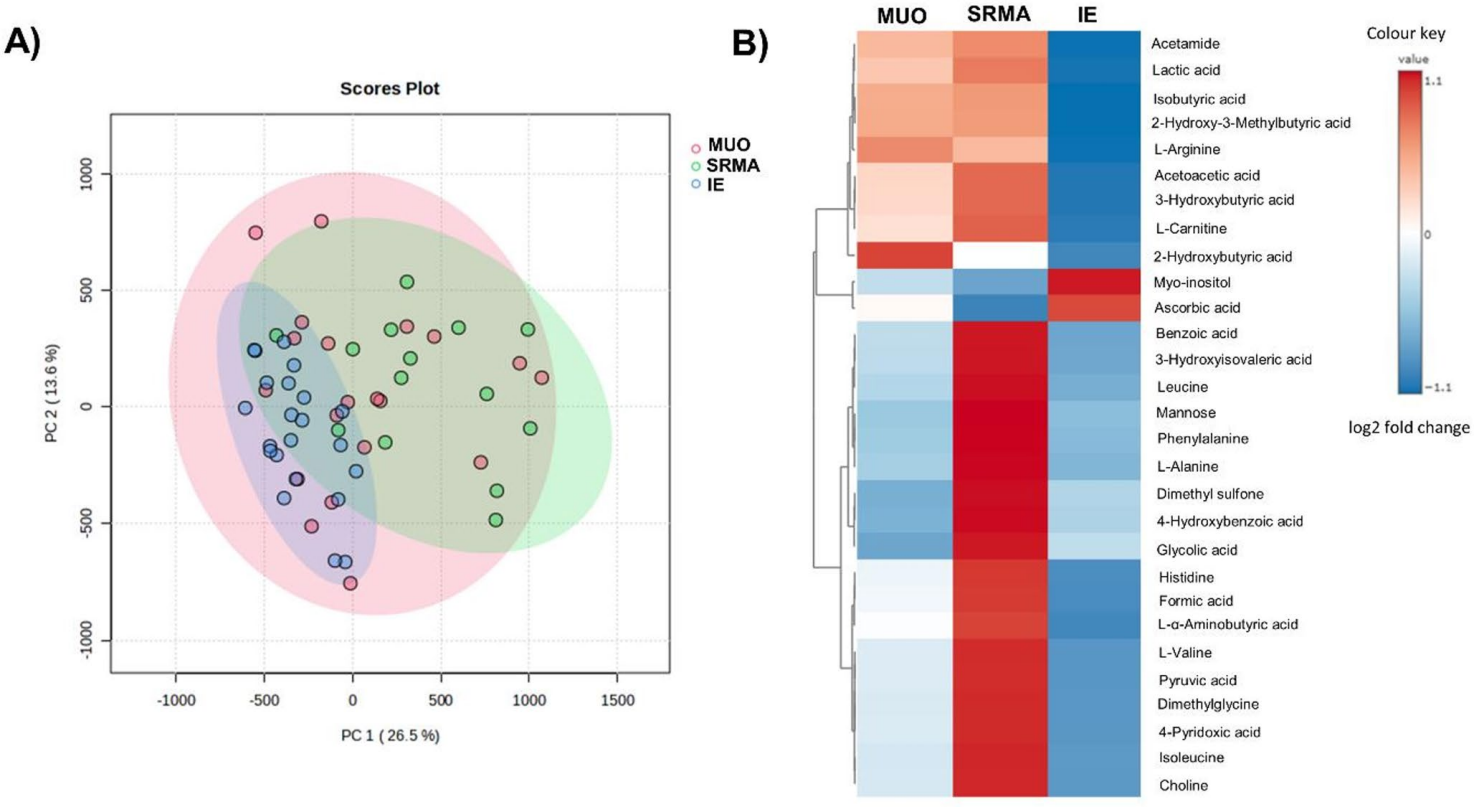
**A** PCA score plot for MUO (red), SRMA (green) and IE (blue) CSF samples. **B** heatmap showing the metabolites showing differential abundance between the disease groups based on *Kruskal Wallis test*

Unsupervised PCA showed prominent overlap between the different groups, with the first two principal components (PC) accounting for 40.1% (PC1 26.5% and PC2 13.6% individually) of the variation in the metabolite concentrations (Fig. 1). The margin of the MUO group was more overlapped with both the SRMA and IE groups, which implies that the overall metabolic characteristics of the CSF in MUO is more diverse. The Kruskal Wallis test including all groups showed that 29 metabolites had differential abundance between at least two groups (Table 1). The predominant differences were between the immune-mediated conditions (MUO and SRMA) and IE.

**Table 1:** Significant metabolites identified on one-way ANOVA and Kruskal Wallis test

Metabolite	FDR *p* value ANOVA	FDR *p* value Kruskal Wallis	MUO vs. SRMA	MUO vs. IE	SRMA vs. IE
2-Hydroxy-3-methylbutyric acid	0.0029	3.42 E-05	-	↑	↑
2-Hydroxybutyric acid	0.0072	0.0043	-	↑	-
3-Hydroxybutyric acid	0.0006	9.11 E-05	-	↑	↑
3-Hydroxyisovaleric	*0.0528*	2.71 E-05	NA	NA	NA
4-Hydroxybenzoic acid	0.0004	0.0074	↓	-	↑
4-Pyridoxic acid	*0.0518*	0.0357	NA	NA	NA
Acetamide	0.0408	0.0074	-	↑	↑
Acetoacetic acid	0.0042	0.0028	-	↑	↑
Ascorbic acid	0.0207	0.0074	-	-	↓
Benzoic acid	0.0408	0.0112	↓	-	↑
Choline	1.01 E-05	0.0004	↓	↑	↑
D-Mannose	1.01 E-05	0.0004	↓	-	↑
Dimethyl sulfone	0.0010	0.0015	↓	-	↑
Dimethylglycine	0.0029	0.0112	↓	-	↑
Formic acid	*0.3930*	0.0039	NA	NA	NA
Glycolic acid	*0.0890*	0.0432	NA	NA	NA
Histidine	0.0029	0.0043	↓	-	↑
Isobutyric acid	0.0017	0.0039	-	↑	↑
Isoleucine	0.0004	0.0008	↓	-	↑
L-Alanine	0.0017	0.0019	↓	-	↑
L-alpha-aminobutyric acid	1.47 E-05	2.30 E-05	↓	↑	↑
L-Arginine	0.0030	9.31 E-05	-	↑	↑
L-Carnitine	0.0171	0.0024	-	↑	↑
L-Valine	2.79 E-06	2.04 E-05	↓	↑	↑
Lactic acid	0.0029	0.0039	-	↑	↑
Leucine	0.0061	0.0112	↓	-	↑
Myo-Inositol	0.0109	0.0015	-	↓	↓
Phenylalanine	0.0217	0.0236	↓	-	↑
Pyruvic acid	0.0004	0.0018	↓	-	↑

*FDR* False-Discovery Rate,* IE* Idiopathic Epilepsy,* MUO* Meningoencephalitis of unknown origin,* SRMA* Steroid Responsive Meningitis-Arteritis

↓ indicates downregulation and ↑ indicates upregulation between the different disease groups analyzed (based on tukeys post hoc analysis)

### Metabolite profiling of MUO and SRMA

**Fig. 2:**
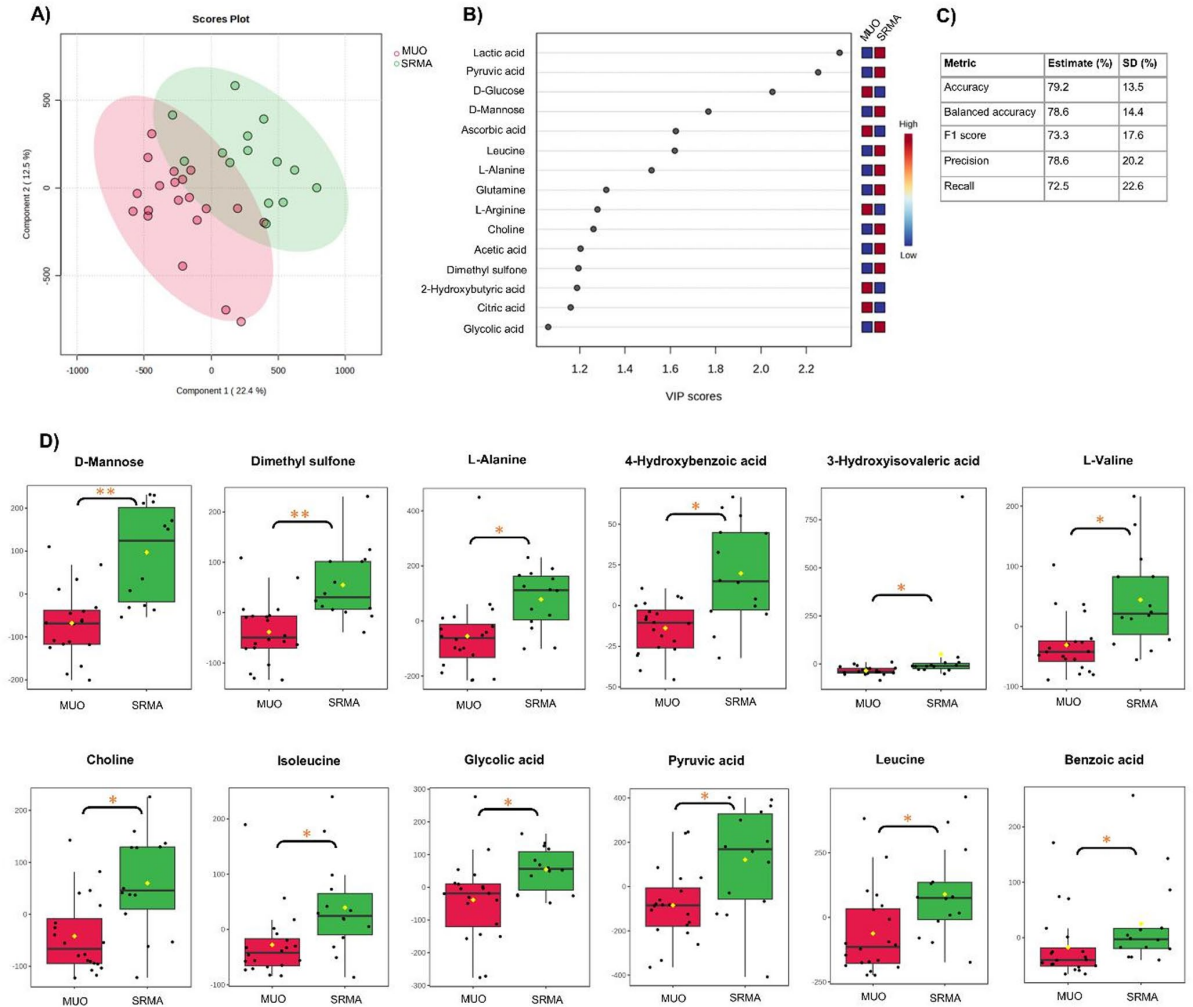
**A** Score plot for the supervised PLS-DA model. **B** VIP scores between MUO and SRMA groups using a 5-component model (R2 = 0.917, Q2 = 0.623). **C** Cross validation metrics. **D** Boxplot of the metabolites showing significant differences between dogs with MUO and SRMA. Asterisks indicate FDR-adjusted p-values < 0.05 (*) or < 0.005 (**)

The PLS-DA model of MUO and SRMA revealed moderate discrimination (balanced accuracy of 78.6%, * R*^*2*^ 0.917) between the groups with several possible discriminators with a VIP score > 1.0 were identified using a 5-component model based on cross-validation (Fig. 2).Univariate analysis between the two groups revealed elevations in levels of *mannose*,*dimethyl sulfone*,*L-Alanine*,*4-hydroxybenzoic acid*,*3-hydroxyisovaleric acid*,*L-valine*,*choline*,*isoleucine*,*glycolic acid*,*pyruvic acid*,*leucine and benzoic acid* in SRMA compared to MUO.

### Metabolite profiling of MUO and IE

**Fig. 3:**
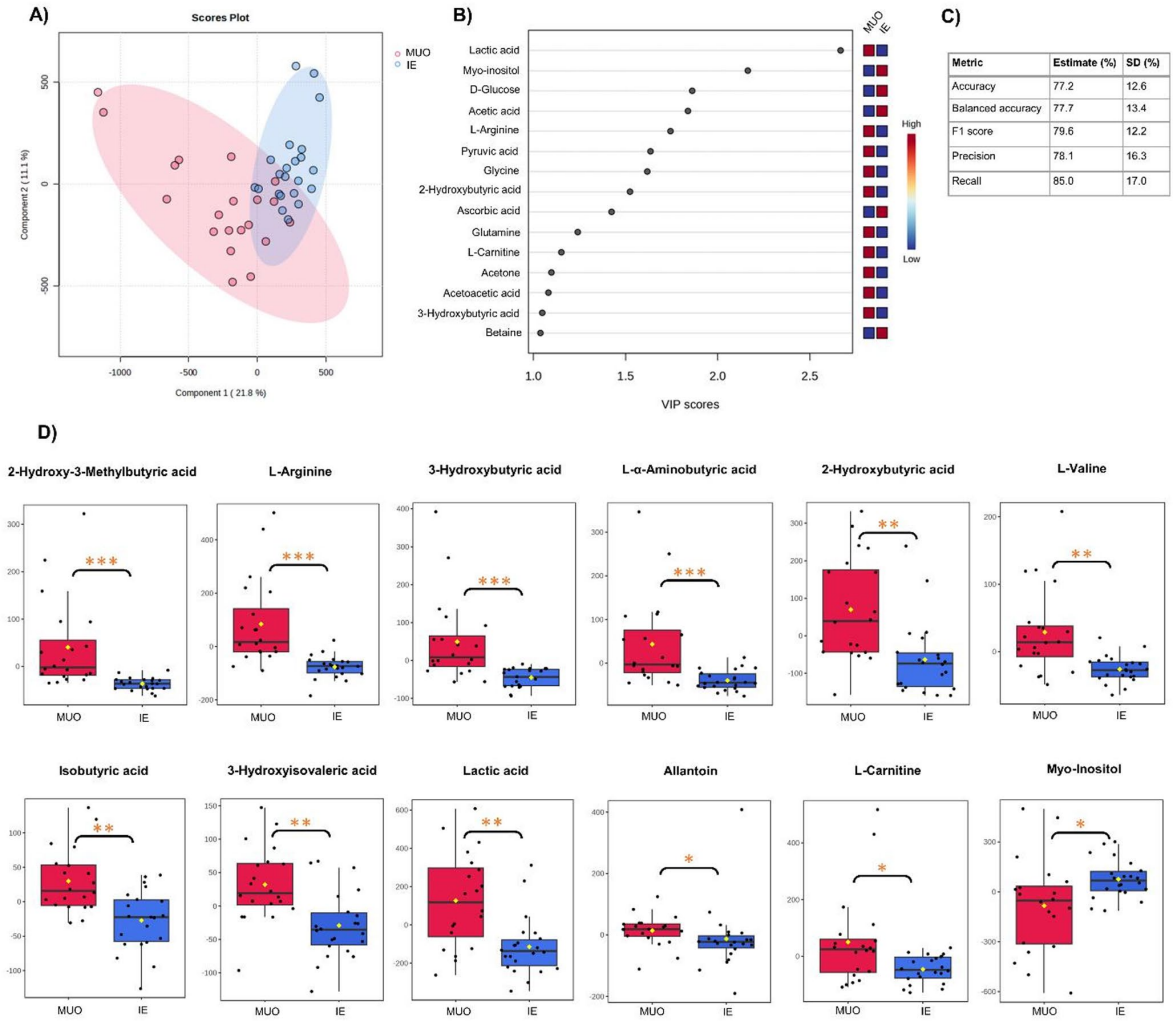
**A** Score plot for the supervised PLS-DA model. **B** VIP scores between MUO and IE groups using a 5-component model (R^2^ = 0.858, Q^2^ = 0.546); **C** Cross validation metrics. **D** Boxplot of metabolites showing significant differences between dogs with MUO and IE. Asterisks indicate FDR-adjusted p-values < 0.05 (*), < 0.005 (**), or < 0.001 (***)

The PLS-DA model of MUO and IE cases also partially discriminated (balanced accuracy of 77.7%, *R*^*2*^ 0.858) between the groups, with several possible discriminatory metabolites were identified (using a 5-component model based on cross-validation) (Fig. 3). Univariate analysis between the two groups revealed elevations in levels of *2-hydroxy-3-methylbutyric acid*,*L-arginine*,*3-hydroxybutyric acid*,*L-α-aminobutyric acid*,*2-hydroxybutyric acid*,*L-valine*,*isobutyric acid*,*3-hydroxyisovaleric acid*,*lactic acid*,*allantoin*,*L-carnitine and decrease in the concentration of myo-inositol*in MUO compared to IE.

###  Metabolite profiling of SRMA and IE

**Fig. 4:**
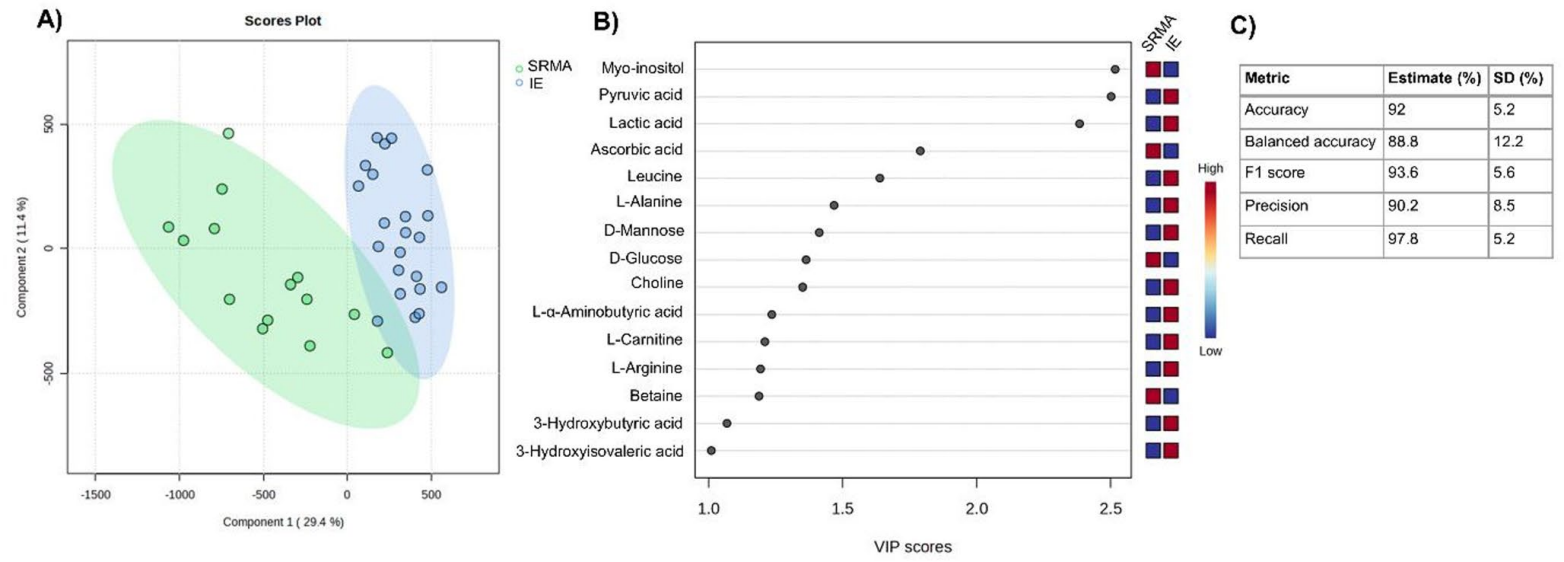
**A** Score plot for the supervised PLS-DA model. **B** VIP scores between SRMA and IE groups using a 4-component model (R^2^ = 0.896, Q^2^ = 0.699); **C** Cross validation metrics

The PLS-DA model of SRMA and IE cases showed higher discrimination (balanced accuracy 88.8%, * R*^*2*^ 0.896) between the groups and several possible discriminatory metabolites were identified (using a 4-component model based on cross-validation) (Fig. 4). Univariate analysis between the two groups revealed elevations in concentrations of *L-valine*,*L-α-aminobutyric acid*,*3-hydroxyisovaleric acid*,*choline*,*isoleucine*,*3-hydroxybutyric acid*,*mannose*,*2-hydroxy-3-methylbutyric acid*,*L-alanine*,*L-carnitine*,*acetoacetic acid*,*pyruvic acid*,*L-arginine*,*formic acid*,* acetamide*,*dimethyl sulfone*,*benzoic acid*,*histidine*,*leucine*,* dimethylglycine*,*phenylalanine*,*lactic acid*,*4-hydroxybenzoic acid*,*4-pyridoxic acid isobutyric acid*,*L-threonine*,*creatine and glycolic acid* and decrease in the concentration of myo-inositol and ascorbic acid in SRMA compared to IE.

### Pathway analysis

**Fig. 5:**
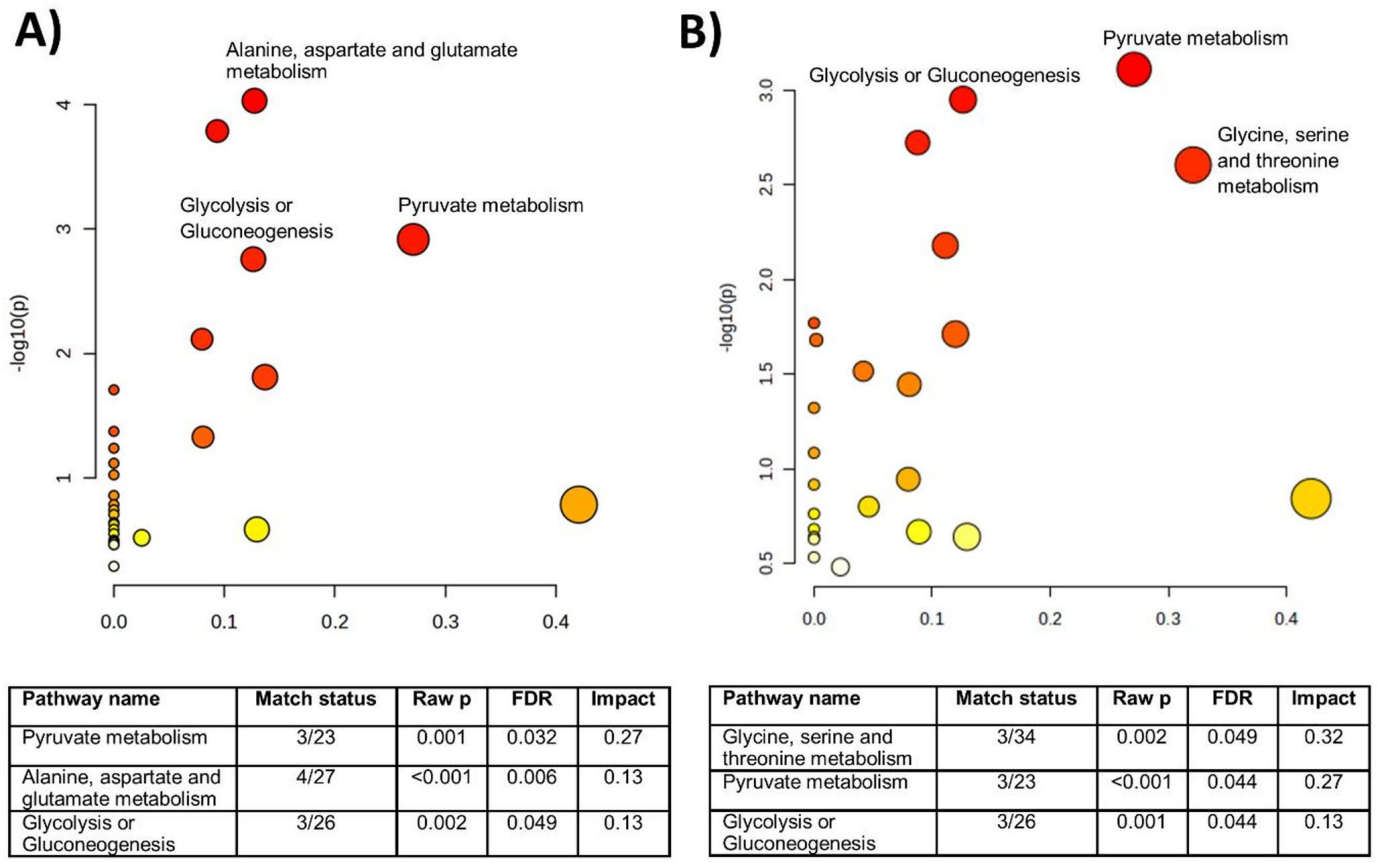
Pathway analysis of key metabolites in CSF samples from (**A**) dogs with MUO and SRMA and (**B**) dogs with MUO and IE. Pathways named in the figure have an impact > 0.1 and an adjusted FDR < 0.05

When evaluating MUO and SRMA, the major relevant pathways were pyruvate metabolism, alanine, aspartate and glutamate metabolism and glycolysis or gluconeogenesis (Fig. 5A). When evaluating MUO and IE, the major relevant pathways were pyruvate metabolism, glycolysis or gluconeogenesis and glycine, serine and threonine metabolism (Fig. 5B).

## Discussion

This study is the first to use ^1^H NMR metabolomics in canine CSF to identify the metabolome changes associated with MUO. Using an untargeted and semi-targeted metabolomic approach, we found several significant differences in the CSF metabolomics of dogs with MUO when compared to IE and SRMA. These findings provide new insights into the metabolic mechanisms in the CSF of dogs with MUO and offer potential diagnostic and therapeutic strategies.

Ideally, this study would compare the CSF metabolome of dogs with MUO with that of healthy controls, but collecting CSF from healthy dogs would not be ethical due to its invasive nature and the need for general anesthesia. We therefore selected two different conditions affecting the CNS in dogs that require CSF analysis for diagnosis, so that excess CSF samples routinely collected during diagnostic testing could be used for comparison; IE served as a non-inflammatory CNS disease group and SRMA provided an inflammatory CNS disease group for comparison. IE is presumed to be genetic in origin and *it is a common neurological complaint in dogs *(Bride et al., 2024). It results in epileptic seizures that occur at varied frequencies and which usually start between 1 and 5 years of age in dogs. It is diagnosed based on the exclusion of gross structural brain disease on MRI and normal CSF analysis, ruling out inflammatory conditions (De Risio, et al., 2015). Although some markers of oxidative stress and inflammation were increased in the plasma of dogs with IE (Verdoodt et al., 2025), all dogs in this study diagnosed with IE had normal CSF cell counts and this condition is typically considered a non-neuroinflammatory disease. SRMA is an immune-mediated disorder affecting the CNS, typically occurring between 6 and 18 months of age in dogs (Wohlsein & Tipold, 2023). It is associated with marked inflammatory changes affecting the CSF, most commonly causing pleocytosis with non-degenerated neutrophils and increased protein content. The characteristic lesion of SRMA is fibrinoid arteritis and leptomeningeal inflammation and associated necrotizing fibrinoid arteritis, typically sparing the brain parenchyma (Tipold & Schatzberg, 2010). These two conditions were selected to try to differentiate the metabolic profile of the different disease states more effectively and explore possible biomarkers.

Several compounds showed significant differences between dogs affected by the different conditions. In general, most metabolites were increased in SRMA and decreased in IE when compared to MUO. Many of these metabolites were amino acids and metabolites involved in energy metabolism, likely reflecting increased energy demand in both inflammatory CNS diseases. Increased concentrations of 3-hydroxybutyric acid and acetoacetic acid in MUO compared to IE suggest an increase in the synthesis and degradation of ketone bodies, likely in response to the elevated energy requirements. L-Carnitine was also observed to be increased in MUO compared to IE and may also reflect its role in the transport of long-chain fatty acids into the mitochondria, for breakdown into acetyl-CoA via β-oxidation; the latter then serves as a substrate for the tricarboxylic acid (TCA) cycle, and ultimately ATP production. Alanine concentration was increased in SRMA compared to MUO but not in MUO compared to IE. This may reflect its possible use as a source for pyruvate production or de novo synthesis of macromolecules within neuronal and immune cells (Li et al., 2007). Notably, alanine can be synthetized from branched-chain amino acids (isoleucine, leucine and valine), all of which were also significantly altered in the CSF. Pyruvate itself was also elevated in SRMA compared to both MUO and IE, suggesting that dysregulation of the TCA cycle may be more important in the pathogenesis of SRMA than in MUO. In contrast, 2-hydroxybutyric acid (2HB) was elevated in MUO compared to IE but no significant changes were noted in relation to SRMA. Increased concentrations of 2HB indicate disrupted homeostasis of the insulin-glucose relationship and this metabolite is considered a biomarker for insulin resistance, during which there is activation of other metabolic pathways (Sousa et al., 2021). Interestingly, insulin resistance has been associated with disease severity in MS (Ruiz-Argüelles et al., 2018) and may also play a role in the pathogenesis of MUO.

Adequate neuronal function is highly dependent on the availability and efficient use of glucose, and dysfunctional glycolysis ultimately leads to cognitive impairment and brain damage (Bhinderwala et al., 2024). Autoimmune disease-related auto-inflammatory responses require large amounts of energy, resulting in increased metabolism of fat, glucose and glutamine and a shift from oxidative phosphorylation to glycolysis (Liu et al., 2025). The reprogramming of energy metabolism in autoimmune diseases likely serves as a pathogenic factor but should also be seen as a potential therapeutic target. Dietary management, as a source of accumulation or elimination of metabolic substrates could be a promising origin for new drug targets (Tancreda et al., 2024).

The only metabolite found to be decreased in both inflammatory CNS conditions compared to IE was myo-inositol, also showing a high VIP value for discriminating between MUO and IE. Myo-inositol plays a vital role in neuronal signalling as second messenger in numerous neurotransmitter systems (Cordoba et al., 1996). It is synthesized mainly de novo from glucose in the brain and is an important part of glycolipids and cell membrane building compounds. Myo-inositol is a glial cell marker implicated in osmoregulation of astrocytes (Brand et al., 1993) and is essential for myelin formation and function (Maeba et al., 2018) and any of these functions may underlie the changes identified. It has also been shown to be increased in human patients with pharmacoresistant epilepsy and could reflect an increase in glial density or astrocyte activation in this population (Pimentel-Silva et al., 2020).

Metabolomic analysis of CSF in human patients with MS has shown widely variable results, often attributed to the heterogeneity of the disease. Despite this, changes connected to energy metabolism pathways remain consistent across both human and animal models, supporting its role in MS pathogenesis (Bhinderwala et al., 2024; Cocco et al., 2016; Kim et al., 2017; Noga et al., 2012; Reinke et al., 2014). These differences in the reported results may be partly due to inclusion of samples from patients with MS in various stages of the disease and often receiving different treatments. It is likely that marked changes in CNS metabolism occur over the course of disease and this can explain the often contradicting results in the individual metabolite levels at different timepoints although the affected pathways remain more consistent (Noga et al., 2012; Židó et al., 2022). Although well-defined disease stages have not yet been defined in MUO, the majority of the dogs included in this study presented in the shortly after the development of clinical signs and were not receiving immunosuppressive medication so our results mostly represent the initial changes seen in this disease.

Pathway analysis has, over the past years, emerged as an invaluable tool to further understand and explore metabolomics data. This study identified that the major pathways altered between MUO and *the other conditions* include pyruvate metabolism, glycolysis or gluconeogenesis, *alanine*,*aspartate and glutamate metabolism* and glycine and serine and threonine metabolism. The different metabolites in these pathways may show potential for further evaluation as biomarkers in MUO diagnosis and treatment and could guide future therapeutic interventions.

This study is exploratory and is limited by the number of CSF samples available for analysis, as often only small quantities are collected from dogs. A possible limitation of this study is the overall limited number of metabolites identified. This is likely due to the use of NMR-based techniques, which although excellent for quantification and reproducibility of the data, are less sensitive than mass spectrometry, requiring higher concentrations of metabolites for their detection (Nagana Gowda & Raftery, 2015). *At this time*,* there are multiple schools of thought about what the most appropriate model choice for analyzing metabolomics data is and different tests are used across different studies; we elected to perform PLS-DA *(Tapp, 2009) but as the data is widely accessible, other investigators could chose to evaluate the data using other models if this is of interest. As the three diseases included most commonly affect dogs of different breeds and ages (with SRMA being typically diagnosed before 18 m of age), this has resulted in marked differences between the group populations, which may have affected the findings. The diagnostic criteria for MUO still vary between studies and need further refinement which may explain the heterogeneity of the MUO population, especially with the inclusion of cases of the suspected different subtypes (Nessler & Tipold, 2024). All dogs were anaesthetized for CSF collection and some were administered different medications (Table S4) prior to CSF collection and this may have also affected the results. Another consideration is that some of the metabolic responses may not be unique to MUO and be observed in other CNS or autoimmune diseases in general.

## Conclusion

In this exploratory study comparing the CSF metabolome of dogs with MUO and those with SRMA and IE, we found several differences in the CSF of dogs affected by these diseases. Pathway analysis revealed that these metabolites were mainly involved in pyruvate metabolism, glycolysis or gluconeogenesis, glycine, serine and threonine metabolism and *alanine*,* aspartate and glutamate metabolism*.

These findings advance our understanding of MUO and can guide in the development of diagnostic and therapeutic strategies for this disease. Many of the altered metabolites were amino acids and metabolites involved in energy metabolism, similarly to what is seen in MS patients and animal models, further supporting the use of MUO as a spontaneous animal model for MS.

## Supplementary Information

Below is the link to the electronic supplementary material.

Supplementary Material 1

## Data Availability

The metabolomics and metadata reported in this paper are available via [https://www.ebi.ac.uk/Metabolights/MTBLS6053] study identifier [MTBLS6053].
