# Supplementary material for: NMR metabolomic profiling of cerebrospinal fluid from dogs with meningoencephalitis of unknown origin demonstrates metabolic similarities to multiple sclerosis
Source: Metabolomics. 2026 Feb 9;22(2):24. doi: 10.1007/s11306-026-02403-x (PMC12886375; doi:10.1007/s11306-026-02403-x)
Supplement: Supplementary file 1 — Supplementary Material 1 [file 11306_2026_2403_MOESM1_ESM.docx]

**
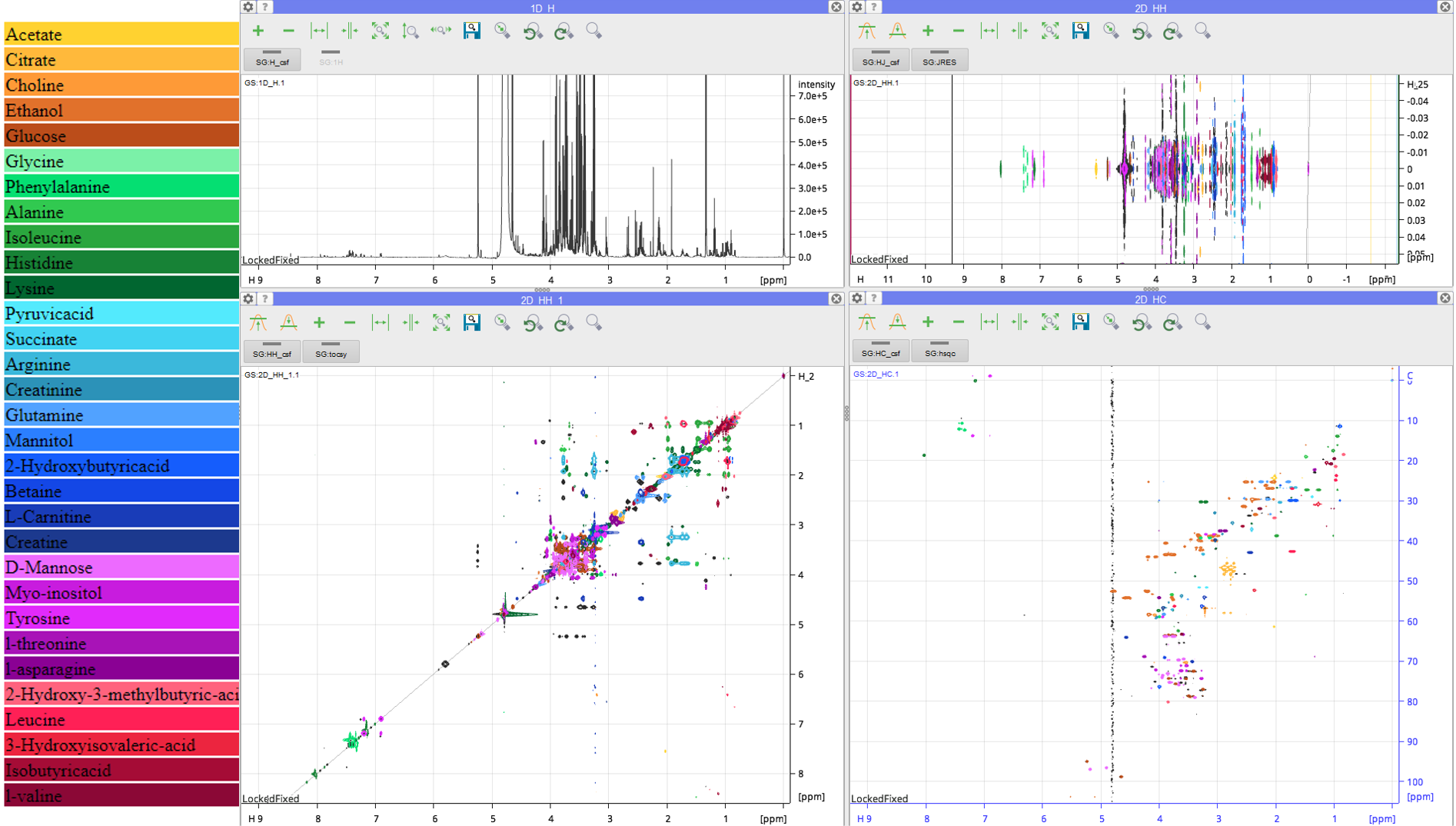
**

**Supplementary Figure S1.** Metabolite Identification via 2-dimensional spectra JRES, HH TOCSY and HSQC standard matching to CSF samples JRES, TOCSY and HSQC in-house library using CcpNmr Analysis Metabolomics


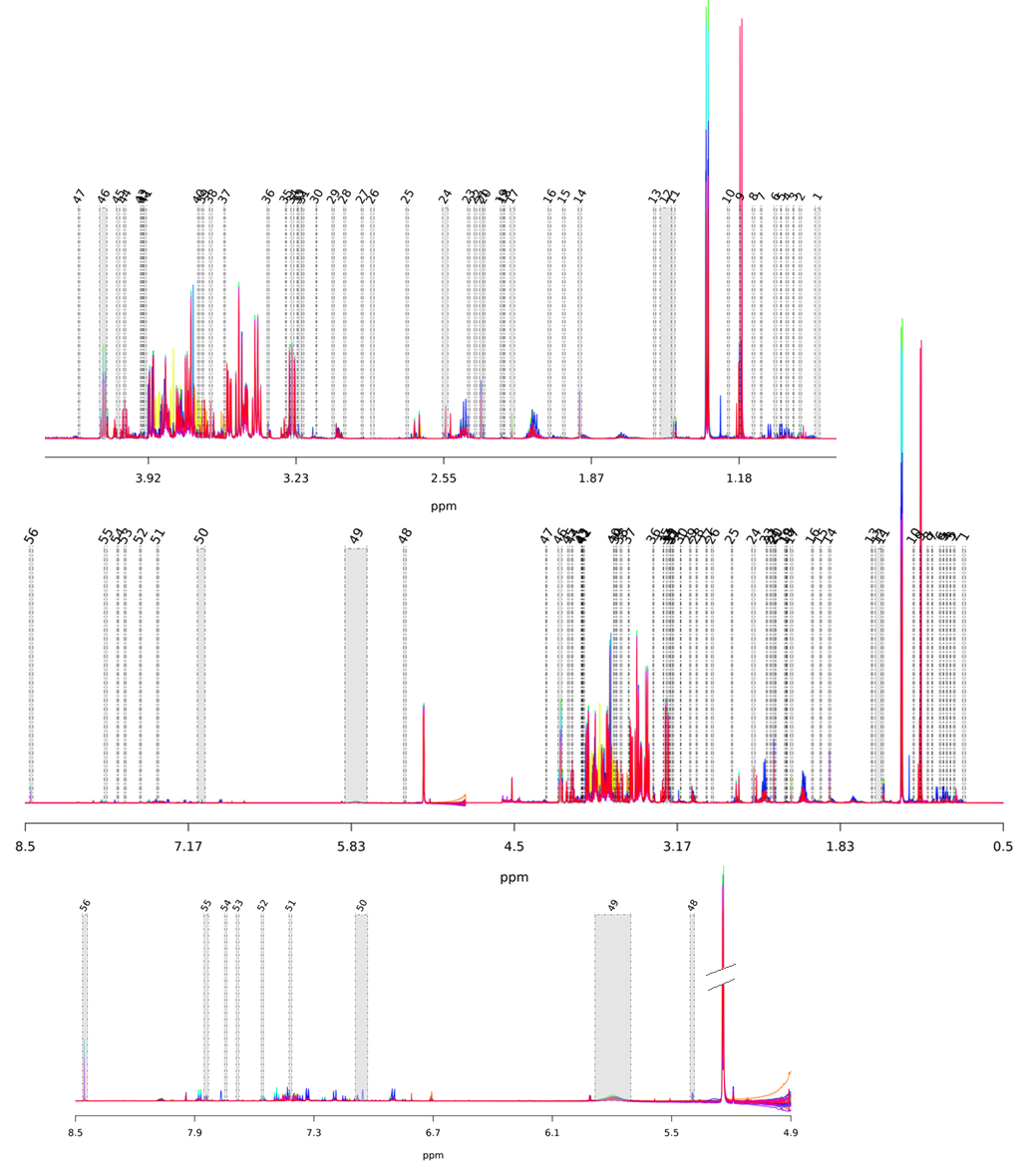
**Supplemental Figure S2. CSF ^1^H CPMG spectra with Selected representative peaks.**  Spectra plotting of entire CSF cohort and bin boundary overlay performed in galaxy.pgb.liv.ac.uk ([https://*github*.com/PGB-LIV/*tameNMR*](https://github.com/PGB-LIV/tameNMR)). Top panel: aliphatic region between 4ppm and 0.7ppm. Middle panel: whole spectrum water region between 4.9 and 4.6ppm has been edited out (replaced with 0 intensities). Bottom panel: aromatic region between 8.5 and 4.9ppm. Metabolite peaks annotated are [1] 2-Hydroxy-3-methylbutyric-acid, [2] 2-Hydroxybutyric-acid, [3] 2-Hydroxy-3-methylpentanoic-acid, [4] L-Leucine, [5] L-Alpha-aminobutyric-acid, [6] L-Isoleucine, [7] (S)-3-Hydroxyisobutyric-acid, [8] Isobutyric-acid, [9] Isopropylalcohol, [10] (R)-3-Hydroxybutyric-acid, [11] L-Alanine, [12] L-Lysine, [13] Butyric-acid, [14] Acetic-acid, [15] Acetamide, [16] N-Acetyl-L-aspartic-acid, [17] Acetone, [18] L-Valine, [19] Acetoacetic-acid, [20] 3-Hydroxyisovaleric-acid, [21] Pyruvic-acid, [22] Succinic-acid, [23] L-Glutamine, [24] Citric-acid, [25] Dimethylamine, [26] L-Asparagine, [27] Dimethylglycine, [28] Oxoglutaric-acid, [29] L-Tyrosine, [30] Dimethylsulfone, [31] Choline, [32] L-Carnitine, [33] L-Arginine, [34] D-Glucose, [35] Betaine, [36] Methanol, [37] Glycine, [38] Myoinositol, [39] Ethanol, [40] Mannitol, [41] Creatine, [42] D-Mannose, [43] Glycolic-acid, [44] Ascorbic-acid, [45] Creatinine, [46] L-Lactic-acid, [47] L-Threonine, [48] Allantoin, [49] Urea, [50] L-Histidine, [51] L-Phenylalanine, [52] Benzoic-acid, [53] Pyridoxine, [54] 4-Hydroxybenzoic-acid, [55] 4-Pyridoxic-acid, [56] Formic-acid.


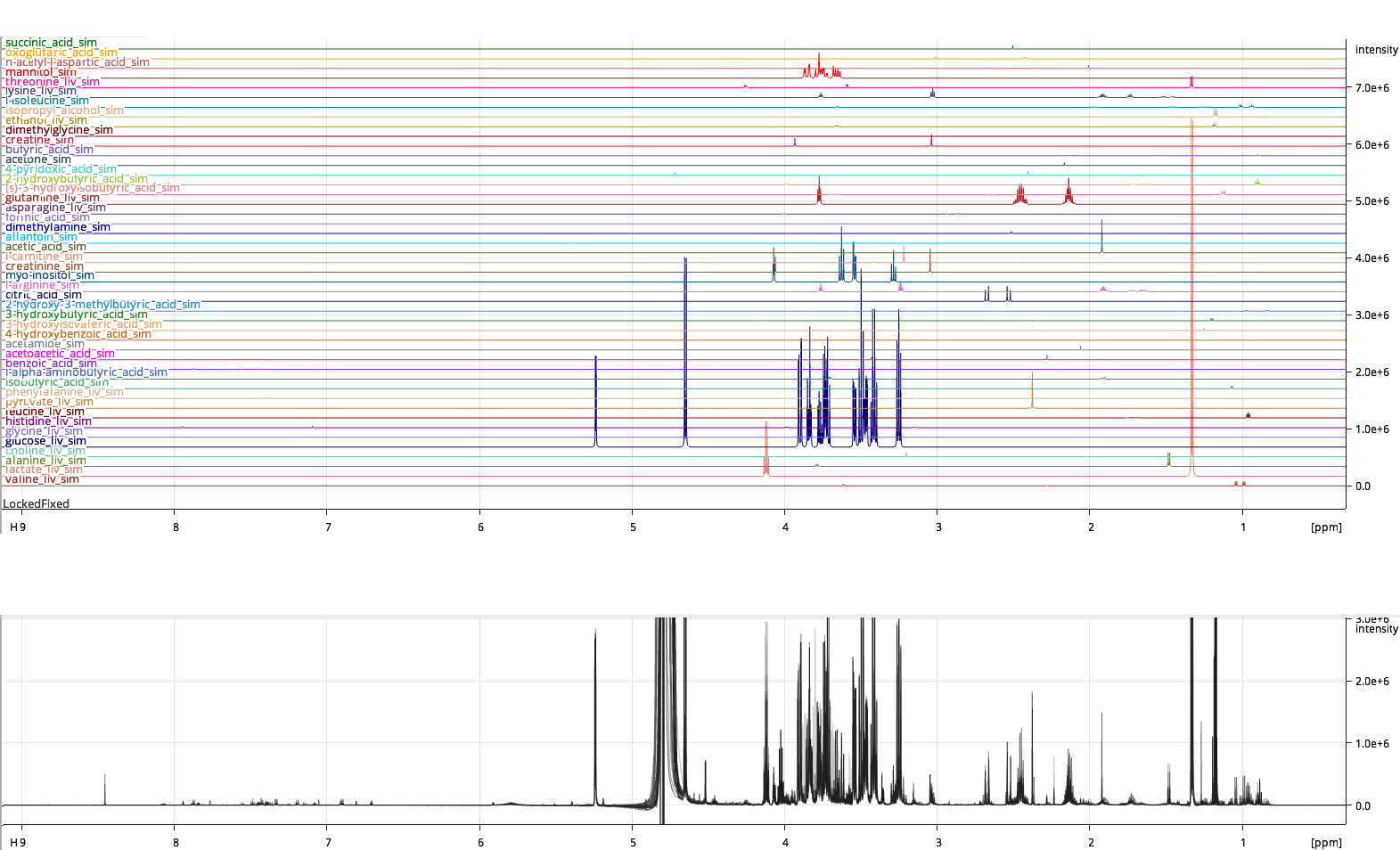


**Supplementary Figure S3. simulated metabolite standards annotated in CSF ^1^H CPMG.** Top panel: Metabolite simulated spectra derived from either in-house standard spectra acquired under identical conditions and equipment or CASMDB library (<https://www.biorxiv.org/content/10.1101/2024.05.05.592402v1>). Bottom panel: CSF ^1^H CPMG over lay of entire cohort plotted in varying grey shades. Spectra plotting and analysis performed within CcpNmr AnalysisMetabolomics software ([www.ccpn.ac.uk](http://www.ccpn.ac.uk/))

**Supplementary Table S1.** Key acquisition parameters for 1D and 2D NMR spectra employed in this study. Full 1D NMR parameter sets are available for download with the raw and processed data used for statistical analysis at ebi.ac.auk/MetaboLights/MTBLS6053

| Dimension | 1D | Pseudo-2D | 2D | |
| --- | --- | --- | --- | --- |
| Parameters | CPMG | JRES | TOCSY | HSQC |
| Pulse Program (Bruker) | cpmgpr1d | jresgpprqf | dipsi2esgpph | hsqcegpsisp.2 |
| Nuclei observed in each dimension | ^1^H | ^1^H , *J*_H_ | ^1^H ,^1^H | ^1^H and ^13^C |
| Number of Scans | 256 | 32 | 8 | 8 |
| Dummy Scans | 4 | 16 | 32 | 16 |
| Temperature | 25 °C | 25 °C | 25 °C | 25 °C |
| Interscan delay (s) | 4 | 2 | 2 | 1.5 |
| CMPG loop & total duration (ms)  TOCSY mixing time (ms) | 128  80 | n/a | 80 | n/a |
| Acquisition Time (s) | 3.0671 | 0.4086 | 0.1219 | 0.0731 |
| Direct Dimension Spectral Width (ppm) | 17.17 | 14.32 | 12.00 | 10.00 |
| Number of Points (Direct Dimension) | 73728 | 8192 | 2048 | 1024 |
| Indirect Dimension Spectral Width (ppm) | NA | 2.45 | 12 | 120.00 |
| Number of Points (Indirect Dimension) | NA | 40 | 512 | 1536 |

**Supplementary Table S2.** Metabolite annotation and identification chemical shift assignment, centre of peak clusters reported in ppm.

| Metabolite ID | Metabolite Name | representative bin | MSI level | Peak multiplet centre for each hydrogen environment (ppm) |
| --- | --- | --- | --- | --- |
| HMDB0000023 | (S)-3-Hydroxyisobutyricacid | 1.2381 to 1.228 | 2 | 1.1236, 2.6490, 3.6831 |
| HMDB0000407 | 2-Hydroxy-3-methylbutyricacid | 1.0854 to 1.0777 | 2 | 0.8336, 0.9700, 2.0306, 3.8525 |
| HMDB0000008 | 2-Hydroxybutyricacid | 0.8333 to 0.8095 | 1 | 0.8998, 1.6409, 1.7344, 3.9905 |
| HMDB0000317 | 2-Hydroxy-3-methylpentanoicacid | 0.935 to 0.9298 | 2 | 3.872, 1.7484, 1.3514, 1.1494, 0.9314, 0.8647 |
| HMDB0000011 | (R)-3-Hydroxybutyricacid | 0.9068 to 0.8955 | 1 | 1.2006, 2.3708, 2.4305, 4.1745 |
| HMDB0000754 | 3-Hydroxyisovalericacid | 2.3686 to 2.3584 | 1 | 1.2503, 2.3657 |
| HMDB0000500 | 4-Hydroxybenzoicacid | 7.7493 to 7.7372 | 2 | 7.7367, 6.9057 |
| HMDB0000017 | 4-Pyridoxicacid | 7.8539 to 7.8309 | 2 | 2.4047, 7.8456, 4.7182 |
| HMDB0031645 | Acetamide | 2.0001 to 1.9882 |  | 2.0608 |
| HMDB0000042 | Acetic acid | 1.9277 to 1.9115 | 1 | 1.9207 |
| HMDB0000060 | Acetoacetic acid | 2.2883 to 2.2776 | 2 | 2.2807, 3.4319 |
| HMDB0001659 | Acetone | 2.2432 to 2.2225 | 2 | 2.1661 |
| HMDB0000462 | Allantoin | 5.407 to 5.3857 | 2 | 5.3923 |
| HMDB0000044 | Ascorbic acid | 4.0316 to 4.0214 | 2 | 3.7429, 3.7188, 4.0264, 4.5203 |
| HMDB0001870 | Benzoic acid | 7.565 to 7.5526 | 2 | 7.5577, 7.4574, 7.8742 |
| HMDB0000043 | Betaine | 3.2822 to 3.2771 | 1 | 3.8780, 3.2370 |
| HMDB0000039 | Butyric acid | 1.5798 to 1.5691 | 2 | 0.8993, 1.5393, 2.1418 |
| HMDB0000097 | Choline | 3.2071 to 3.1969 | 1 | 3.5261, 4.0678, 3.2033 |
| HMDB0000094 | Citric acid | 2.5573 to 2.5305 | 1 | 2.6741, 2.5323 |
| HMDB0000064 | Creatine | 3.9362 to 3.9256 | 1 | 3.0376, 3.9336 |
| HMDB0000562 | Creatinine | 4.0641 to 4.0491 | 1 | 3.0469, 4.0613 |
| HMDB0000122 | D-Glucose | 3.2579 to 3.2431 | 1 | 5.2382, 4.6507, 3.9001, 3.8394, 3.7694, 3.7332, 3.7188, 3.5427, 3.4988, 3.4699, 3.4208, 3.4101, 3.2519 |
| HMDB0000087 | Dimethylamine | 2.7246 to 2.7138 | 1 | 2.5135 |
| HMDB0000092 | Dimethylglycine | 2.9318 to 2.923 | 2 | 2.9211, 3.7106, 3.7176 |
| HMDB0004983 | Dimethyl sulfone | 3.1413 to 3.137 | 2 | 3.1413 |
| HMDB0000169 | D-Mannose | 3.944 to 3.9397 | 2 | 5.1751, 4.8952, 3.9377, 3.9267, 3.9008, 3.8674, 3.8405, 3.8059, 3.75703, 3.7265, 3.6516, 3.6516, 3.5658, 3.374 |
| HMDB0000108 | Ethanol | 3.6691 to 3.66 | 1 | 3.6586, 1.1850 |
| HMDB0000142 | Formic acid | 8.4649 to 8.4396 | 2 | 8.4554 |
| HMDB0000123 | Glycine | 3.5674 to 3.5604 | 1 | 3.5562 |
| HMDB0000115 | Glycolic acid | 3.954 to 3.9481 | 2 | 3.9338 |
| HMDB0001873 | Isobutyric acid | 1.1231 to 1.1115 | 1 | 1.0691, 2.3702 |
| HMDB0000863 | Isopropyl alcohol | 1.1823 to 1.1768 | 2 | 1.1750, 4.0124 |
| HMDB0000161 | L-Alanine | 1.4969 to 1.48 | 1 | 1.4813, 3.7868 |
| HMDB0000452 | L-Alpha-aminobutyric acid | 0.9912 to 0.9881 | 2 | 3.7034, 1.9234 |
| HMDB0000517 | L-Arginine | 3.2296 to 3.2238 | 1 | 1.6521, 1.7209, 1.9024, 1.9201, 3.2366, 3.2426, 3.7639 |
| HMDB0000168 | L-Asparagine | 2.8882 to 2.8721 | 1 | 4.0017, 2.9493, 2.8609 |
| HMDB0000062 | L-Carnitine | 3.2238 to 3.2071 | 1 | 3.2190, 2.4322, 2.4761, 3.4209, 3.4052, 4.5578 |
| HMDB0000641 | L-Glutamine | 2.4397 to 2.4291 | 1 | 3.7739, 2.4736, 2.4363, 2.1528, 2.1220 |
| HMDB0000177 | L-Histidine | 7.0917 to 7.0298 | 1 | 7.9467, 7.0945, 3.9898, 3.2472, 3.1473 |
| HMDB0000172 | L-Isoleucine | 1.0223 to 1.0096 | 1 | 0.9391, 1.4576, 1.2499, 1.9705, 1.0101, 3.6575 |
| HMDB0000190 | L-Lactic acid | 4.1427 to 4.1092 | 1 | 4.1204, 1.3303 |
| HMDB0000687 | L-Leucine | 0.9665 to 0.9559 | 1 | 3.7409, 1.7221, 0.9612 |
| HMDB0000182 | L-Lysine | 1.5489 to 1.4969 | 1 | 3.0304, 3.7630, 1.9149, 1.7360, 1.5199, 1.4550 |
| HMDB0000159 | L-Phenylalanine | 7.4246 to 7.4109 | 1 | 4.0000, 3.2863, 3.1395, 7.4315, 7.3834, 7.4866 |
| HMDB0000167 | L-Threonine | 4.2418 to 4.2346 | 1 | 3.5907, 1.3330, 4.2572 |
| HMDB0000158 | L-Tyrosine | 3.0667 to 3.0559 | 1 | 7.1941, 6.9033, 3.9373, 3.2049, 3.0564 |
| HMDB0000883 | L-Valine | 2.2722 to 2.2676 | 1 | 3.6155, 2.2787, 1.0411, 0.9895, 1.0101, 3.6575 |
| HMDB0000765 | Mannitol | 3.6882 to 3.6799 | 2 | 3.8634, 3.7935, 3.7506, 3.6726 |
| HMDB0001875 | Methanol | 3.3671 to 3.3582 | 2 | 3.3400 |
| HMDB0000211 | Myoinositol | 3.6344 to 3.6205 | 1 | 3.2869, 3.6273, 3.6267, 3.5434, 3.5432, 4.0717 |
| HMDB0000812 | N-Acetyl-L-aspartic acid | 2.0673 to 2.0544 | 2 | 2.0057, 4.3792, 2.6687, 2.4869 |
| HMDB0000208 | Oxoglutaric acid | 3.0134 to 3.0034 | 2 | 3.0200, 2.4150 |
| HMDB0000239 | Pyridoxine | 7.6913 to 7.6769 | 2 | 2.4505, 7.6845, 4.8108, 4.7283 |
| HMDB0000243 | Pyruvic acid | 2.3846 to 2.3686 | 1 | 2.3765 |
| HMDB0000254 | Succinic acid | 2.4094 to 2.3977 | 1 | 2.5057 |
| HMDB0000294 | Urea | 5.8858 to 5.705 | 2 | 5.7684 |

**Supplementary Table S3.** Demographic and clinical characteristics of the 56 dogs included in the study

|  | **MUO (n=20)** | **SRMA (n=14)** | **IE (n=22)** |
| --- | --- | --- | --- |
| Median age (IQR) | 45 months (30-71) | 11 months (8-13) | 44 months (24-59) |
| Median bodyweight | 8 kg (3-13) | 11 kg (9-16) | 18 kg (12-24) |
| Sex and neuter status | 9 F (5 neutered)  11 M (5 neutered) | 9 F (3 neutered)  5 M (2 neutered) | 2 F (2 neutered)  20 M (8 neutered) |
| CSF collection site | 13 cervical  7 lumbar | 14 cervical | 21 cervical  1 lumbar |
| Median CSF TNCC | 18 (9-82) | 210 (87-342) | 1 (0-1) |
| Median CSF protein | 0.46 (0.3-0.78) | 0.4 (0.33-0.82) | 0.18 (0.16-0.2) |

CSF, Cerebrospinal fluid; F, Female; IE, Idiopathic Epilepsy; IQR, Interquartile range; M, Male; MUO, Meningoencephalitis of unknown origin; SRMA, Steroid Responsive Meningitis-Arteritis; TNCC, total nucleated cell count

**Supplementary Table S4.** List of medication administered prior to CSF collection in the 56 dogs included in the study.

| Treatments administered prior to CSF collection | Number of dogs (n=56) |
| --- | --- |
| Acepromazine | n=1 (MUO=1) |
| Alfaxalone | n=2 (MUO=1, IE=1) |
| Buprenorphine | n=3 (SRMA=3) |
| Butorphanol | n=43 (MUO=18, SRMA=3, IE=22) |
| Cefalexin | n=1 (MUO=1) |
| Dexamethasone | n=2 (MUO=2) |
| Gabapentin | n=8 (MUO=2, SRMA=6) |
| Imepitoin | n=4 (MUO=1, IE=3) |
| Ketamine | n=4 (MUO=1, SRMA=2, IE=1) |
| Levetiracetam | n=4 (MUO=1, IE=3) |
| Lidocaine | n=6 (MUO=5, IE=1) |
| Mannitol | n=6 (MUO=6) |
| Medetomidine | n=56 |
| Meloxicam | n=8 (MUO=5, SRMA=2, IE=1) |
| Methadone | n=9 (MUO=2, SRMA=7) |
| Metronidazole | n=1 (MUO=1) |
| Midazolam | n=4 IE=4) |
| Paracetamol | n=14 (MUO=3, SRMA=11) |
| Phenobarbital | n=9 (MUO=2, IE=7) |
| Potentiated amoxicillin | n=1 (SRMA=1) |
| Propofol | n=54 (MUO=19, SRMA=14, IE=21) |
